# Advancing Age Inclusivity in a Pandemic: Age-Friendly University (AFU) Campuses Take Action

**DOI:** 10.1093/geroni/igab046.1493

**Published:** 2021-12-17

**Authors:** Joann Montepare, Kimberly Farah

## Abstract

The COVID-19 pandemic presented extraordinary challenges for professionals in the aging field across campuses and communities, calling for rethinking and redesigning how their work was structured, their programs were delivered, and their connections were sustained. The pandemic also made clear the value of being an age-friendly institution of higher education, especially as we experience historic changes in age demographics. This symposium features campus leaders representing institutional partners of the Age-Friendly University (AFU) global initiative (endorsed by GSA’s Academy for Gerontology in Higher Education) who will discuss how their age-friendly programs were adapted during the pandemic to continue to advance age inclusivity. These diverse responses exemplify the vast potential of age-friendly opportunities. June and Andreoletti (Central Connecticut State University) will discuss how the Scholars for Life! program supported the engagement of older learners in the neighboring community through the engagement of faculty. Elfenbein (University of North Georgia) will describe how learning experiences for older learners and intergenerational exchange were created beyond the classroom through the Personal Enrichment, Action and Knowledge (PEAK) program. Terhune (Northern Kentucky University) will describe how student support practices and services were adapted to provide working adult students with guidance for navigating their educational needs during the pandemic. Kheirbek (University of Maryland, Baltimore) will describe how age-friendly collaborations with the institution’s medical school leveraged intergenerational connections and technology to foster social connection for hospitalized older adults. Gautam and Melillo (UMass Lowell) discuss how a campus partnership with the Learning in Retirement Association (LIRA) adapted efforts around healthy aging.

